# The genome sequence of common knotgrass, *Polygonum aviculare *L. (Polygonaceae)

**DOI:** 10.12688/wellcomeopenres.21001.1

**Published:** 2024-03-01

**Authors:** Maarten J. M. Christenhusz, Peter M. Hollingsworth, Royal Botanic Gardens Kew Genome Acquisition Lab, Plant Genome Sizing collective, Darwin Tree of Life Barcoding collective, Wellcome Sanger Institute Tree of Life Management, Samples and Laboratory team, Wellcome Sanger Institute Scientific Operations: Sequencing Operations, Wellcome Sanger Institute Tree of Life Core Informatics team, Tree of Life Core Informatics collective, Darwin Tree of Life Consortium

**Affiliations:** 1 Royal Botanic Gardens Kew, Richmond, England, UK; 2 Curtin University, Perth, Western Australia, Australia; 3 Royal Botanic Garden Edinburgh, Edinburgh, Scotland, UK

**Keywords:** Polygonum aviculare, common knotgrass, genome sequence, chromosomal, Caryophyllales

## Abstract

We present a genome assembly from an individual *Polygonum aviculare* (common knotgrass; Eudicot; Magnoliopsida; Caryophyllales; Polygonaceae). The genome sequence is 351.6 megabases in span. Most of the assembly is scaffolded into 10 chromosomal pseudomolecules. The mitochondrial and plastid genome assemblies have lengths of 333.39 kilobases and 163.28 kilobases in length, respectively.

## Species taxonomy

Eukaryota; Viridiplantae; Streptophyta; Streptophytina; Embryophyta; Tracheophyta; Euphyllophyta; Spermatophyta; Magnoliopsida; Mesangiospermae; eudicotyledons; Gunneridae; Pentapetalae; Caryophyllales; Polygonaceae; Polygonoideae; Polygoneae; *Polygonum*; *Polygonum aviculare* L., 1753 (NCBI:txid137693).

## Background

The genome of the common knotgrass, *Polygonum aviculare* L., was sequenced as part of the Darwin Tree of Life Project, a collaborative effort to sequence all named eukaryotic species in the Atlantic Archipelago of Britain and Ireland.

*Polygonum aviculare* is a procumbent to scrambling herbaceous annual with creeping stems up to 2 m long ( Stace *et al.*, 2019; Styles, 1962), but usually shorter. The stems are nodose, with an ochrea sheathing the stem in the axil of the lanceolate leaf. The five petals are white with a green midvein and often a pink tinge on the outside. The flowers are hermaphrodite and small, and considered to be largely self-pollinating ( Styles, 1962). It has five to eight stamens and three fused carpels. The fruit is a dark brown, three-edged nut with a single seed that needs light to germinate. Seeds can lay dormant in the soil seedbank for decades ( Courtney, 1968). The seeds were previously collected and consumed as porridge by Germanic peoples of western Europe ( Nielsen *et al.*, 2021). In herbal medicine, dried knotgrass is sometimes used in concoctions to treat gingivitis or other minor infections of the mouth or throat. It is also suggested as an alternative treatment for breast cancer ( Habibi *et al.*, 2011), but further study on the effective compounds is needed.

Despite its name, common knotgrass is not a grass, but a member of the buckwheat family, Polygonaceae. *Polygonum* is derived from Greek *poly* (many) and *gonu* (knee-joint), referring to the swollen stem nodes. The epithet *aviculare* refers to small birds, said to consume the seeds. They are indeed the favourite food of partridges ( Middleton & Chitty, 1937), among other birds.

It is widely distributed in Europe, North Africa and Asia, with populations as far south as Ethiopia and Myanmar ( POWO, 2023), and it is common throughout Britain and Ireland with the exception of far north-western Scotland and the Scottish Highlands ( Stroh *et al.*, 2023). It has been extensively introduced outside its native range (North and South America, southern Africa, Tasmania, New Zealand), where it can be a colonising weed of fields, gardens and ruderal places ( Meerts *et al.*, 1998; POWO, 2023; Styles, 1962).

In Britain and Ireland it is found on open and disturbed ground, such as arable and other disturbed or trampled land, both rural and urban, soil, manure and waste heaps, paths and tracks, and has been recorded at elevations up to 550 m (with an outlier record of 670 m; Stroh *et al.*, 2023). It can be a notorious weed in gardens, and can be problematic as an agricultural weed, particularly in open and spring-sown crops, such as spring beans, sugar beet, kale, linseed and potatoes ( AHDB, 2008).

The taxonomy of *Polygonum aviculare* is complex, with *Polygonum aviculare* previously treated as an aggregate including taxa now recognised as distinct species such as *P. boreale* (Lange) Small ( Stroh *et al.*, 2023; Styles, 1962). Currently, three subspecies are accepted ( POWO, 2023): *P. aviculare* subsp. *buxiforme* (Small) Costea & Tardif native to North America, *P. aviculare* subsp. *neglectum* (Besser) Arcang. in Siberia, the Caucasus, Greece, Italy and France, and the nominal, widespread *P. aviculare* subsp. *aviculare*, which is sampled here. However, some of these subspecies may also turn out to be independent species. More taxonomic study is needed.

From Britain and Ireland there are chromosome counts from four populations, with a reported chromosome number of 2 *n* = 60 ( Gornall & Bailey, 1993; Styles, 1962). Based on chromosome counts of 2 *n* = 20 and 2 *n* = 40 from other species in the genus, the inferred ploidy of *Polygonum aviculare* is hexaploid ( Meerts *et al.*, 1998; Styles, 1962). A previous isozyme study showed high levels of fixed heterozygosity in *Polygonum aviculare* consistent with the suggestion that it was an allopolyploid ( Meerts *et al.*, 1998). 

In this paper we present a high-quality reference genome as a foundation resource for future studies on taxonomy, agronomy and potential medicinal applications of this herb.

## Genome sequence report

**Figure 1.**
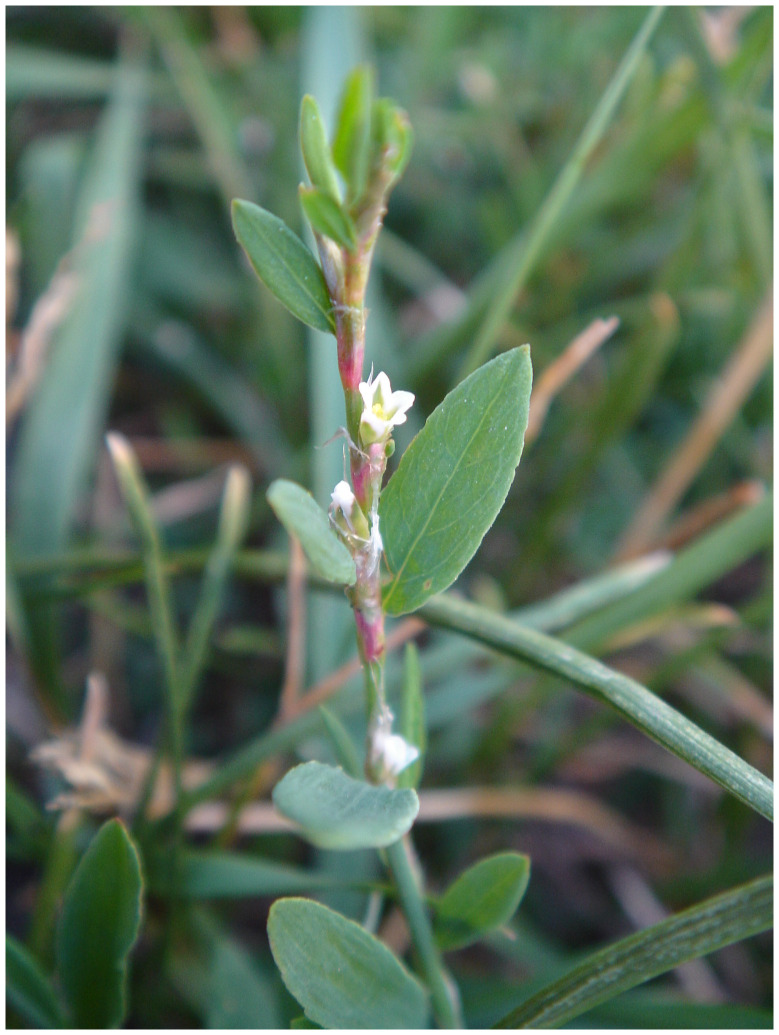
Photograph of the *Polygonum aviculare* (dcPolAvic1) specimen used for genome sequencing (copyright Maarten Christenhusz).

The genome was sequenced from a specimen of *Polygonum aviculare* ( Figure 1) collected from the Royal Botanic Gardens Kew (51.48, –0.29). Using flow cytometry, the genome size (1C-value) was estimated to be 0.93 pg, equivalent to 910 Mb. A total of 68-fold coverage in Pacific Biosciences single-molecule HiFi long reads and 51-fold coverage in 10X Genomics read clouds was generated. Primary assembly contigs were scaffolded with chromosome conformation Hi-C data. Manual assembly curation corrected 5 missing joins or mis-joins and removed 9 haplotypic duplications, reducing the assembly length by 8.16% and the scaffold number by 10.42%, and increasing the scaffold N50 by 5.85%.

**Figure 2.**
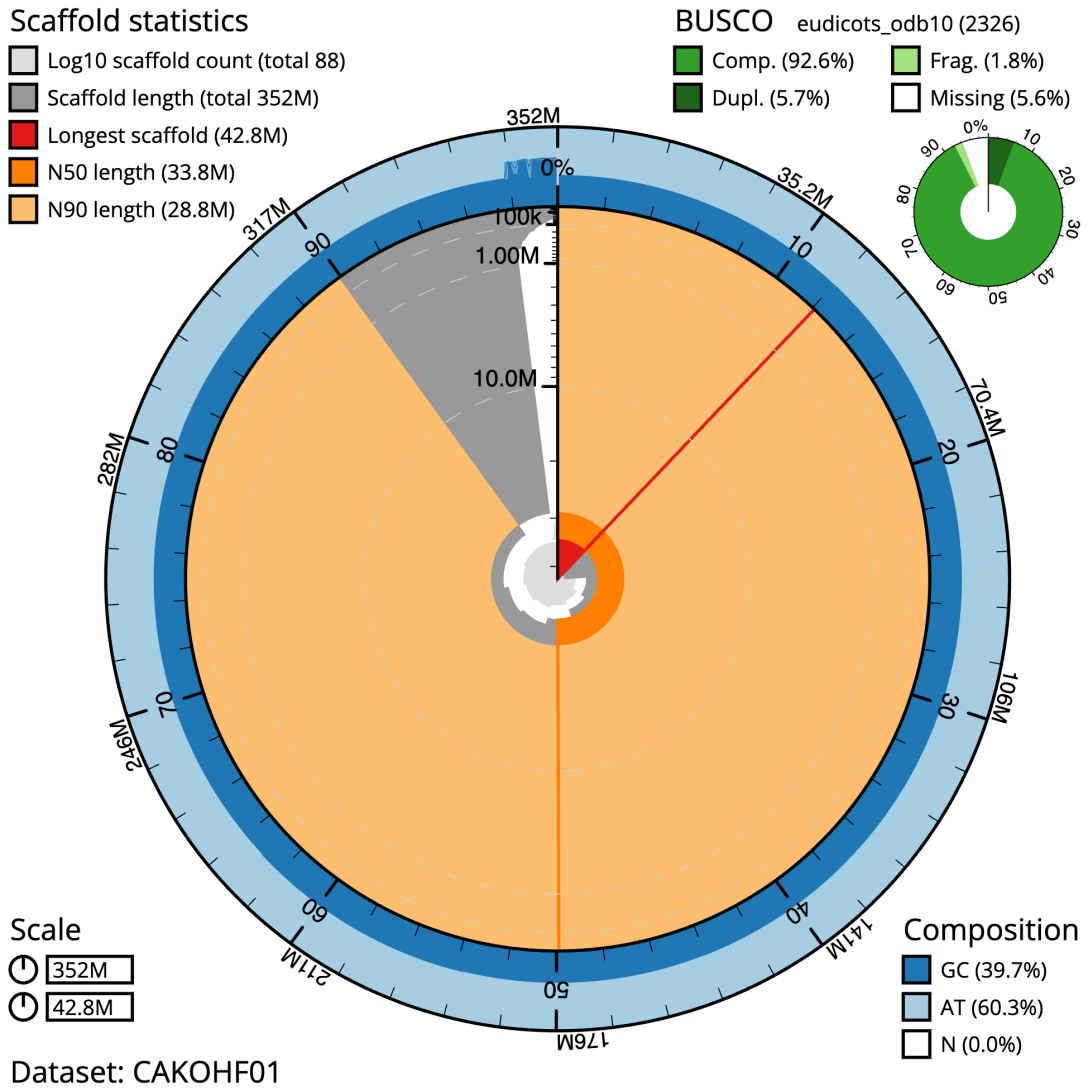
Genome assembly of *Polygonum aviculare*, dcPolAvic1.1: metrics. The BlobToolKit Snailplot shows N50 metrics and BUSCO gene completeness. The main plot is divided into 1,000 size-ordered bins around the circumference with each bin representing 0.1% of the 352,071,428 bp assembly. The distribution of scaffold lengths is shown in dark grey with the plot radius scaled to the longest scaffold present in the assembly (42,788,684 bp, shown in red). Orange and pale-orange arcs show the N50 and N90 scaffold lengths (33,827,450 and 28,844,451 bp), respectively. The pale grey spiral shows the cumulative scaffold count on a log scale with white scale lines showing successive orders of magnitude. The blue and pale-blue area around the outside of the plot shows the distribution of GC, AT and N percentages in the same bins as the inner plot. A summary of complete, fragmented, duplicated and missing BUSCO genes in the eudicots_odb10 set is shown in the top right. An interactive version of this figure is available at https://blobtoolkit.genomehubs.org/view/CAKOHF01/dataset/CAKOHF01/snail.

**Figure 3.**
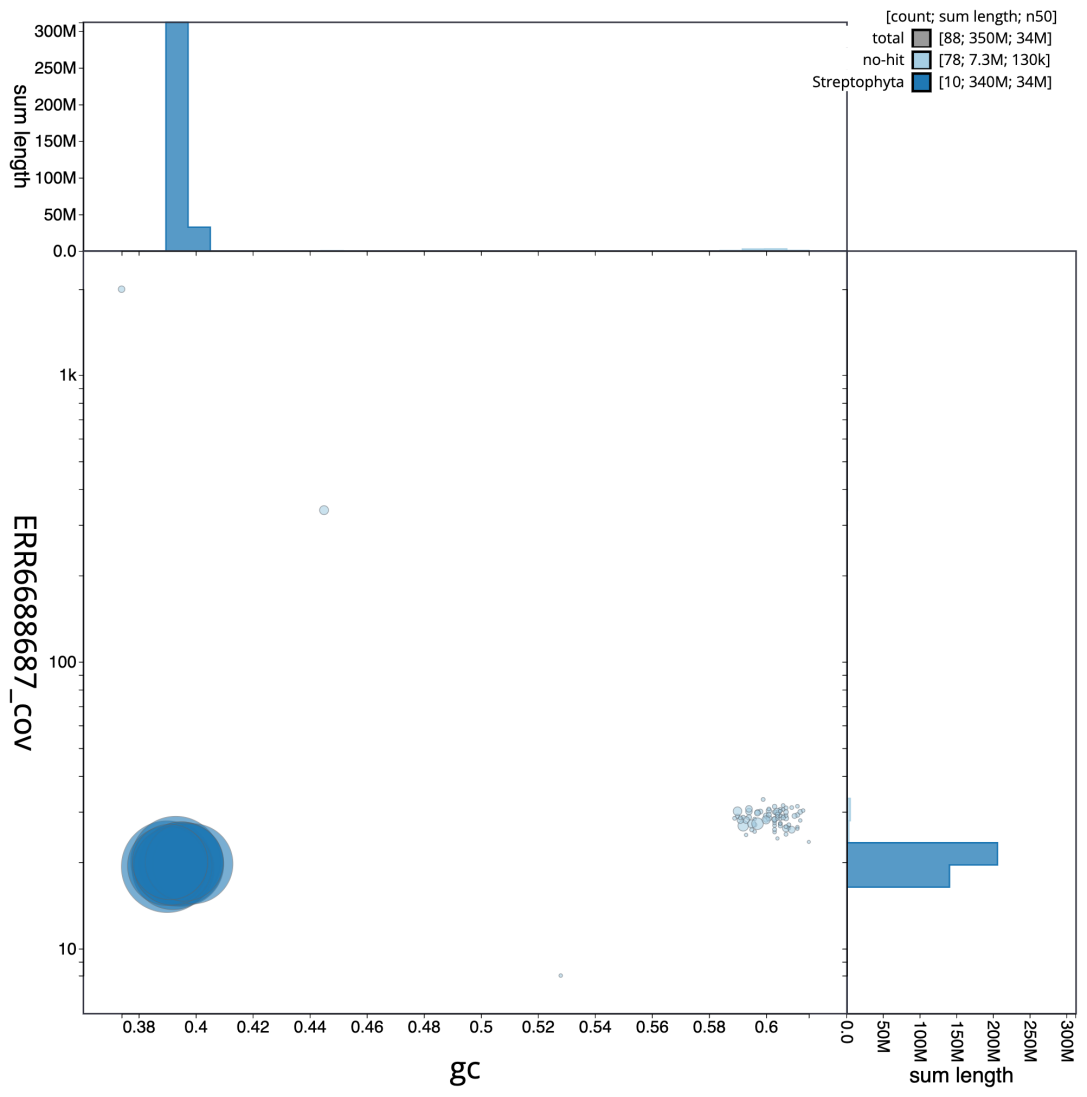
Genome assembly of *Polygonum aviculare*, dcPolAvic1.1: BlobToolKit GC-coverage plot. Scaffolds are coloured by phylum. Circles are sized in proportion to scaffold length. Histograms show the distribution of scaffold length sum along each axis. An interactive version of this figure is available at https://blobtoolkit.genomehubs.org/view/CAKOHF01/dataset/CAKOHF01/blob.

**Figure 4.**
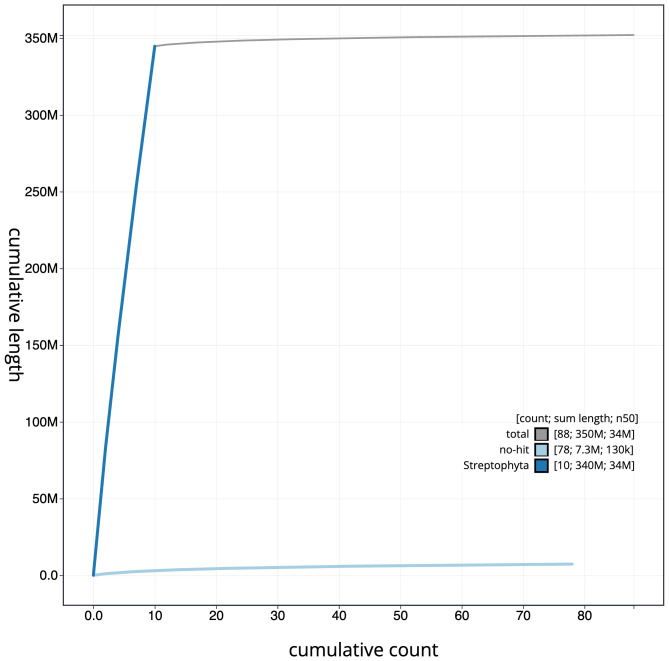
Genome assembly of *Polygonum aviculare*, dcPolAvic1.1: BlobToolKit cumulative sequence plot. The grey line shows cumulative length for all scaffolds. Coloured lines show cumulative lengths of scaffolds assigned to each phylum using the buscogenes taxrule. An interactive version of this figure is available at https://blobtoolkit.genomehubs.org/view/CAKOHF01/dataset/CAKOHF01/cumulative.

**Figure 5.**
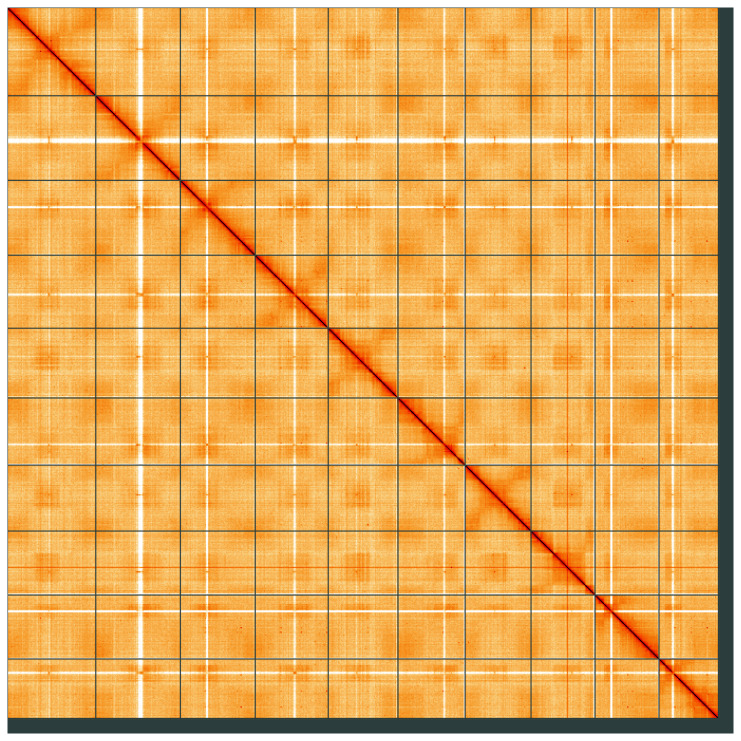
Genome assembly of *Polygonum aviculare*, dcPolAvic1.1: Hi-C contact map of the dcPolAvic1.1 assembly, visualised using HiGlass. Chromosomes are shown in order of size from left to right and top to bottom. An interactive version of this figure may be viewed at https://genome-note-higlass.tol.sanger.ac.uk/l/?d=FBNFeAZsTxm1pwpnccXwTg.

The final assembly has a total length of 351.6 Mb in 86 sequence scaffolds with a scaffold N50 of 33.8 Mb ( Table 1). The discrepancy between the genome assembly size, which is approximately a third of the flow cytometry estimate (above), suggests the individual sequenced here is a hexaploid with an estimated haploid genome size 303 Mb comprising 10 chromosomes. The snailplot in Figure 2 provides a summary of the assembly statistics, while the distribution of assembly scaffolds on GC proportion and coverage is shown in Figure 3. The cumulative assembly plot in Figure 4 shows curves for subsets of scaffolds assigned to different phyla. Most (98.06%) of the assembly sequence was assigned to 10 chromosomal-level scaffolds. Chromosome-scale scaffolds confirmed by the Hi-C data are named in order of size ( Figure 5; Table 2). While not fully phased, the assembly deposited is of one haplotype. Contigs corresponding to a second haplotype have also been deposited. The mitochondrial and plastid genomes were also assembled and can be found as contigs within the multifasta file of the genome submission.

**Table 1.** Genome data for *Polygonum aviculare*, dcPolAvic1.1.

Project accession data		
Assembly identifier	dcPolAvic1.1	
Species	*Polygonum aviculare*	
Specimen	dcPolAvic1	
NCBI taxonomy ID	137693	
BioProject	PRJEB47313	
BioSample ID	SAMEA7522628	
Isolate information	dcPolAvic1: leaf (DNA, RNA and Hi-C sequencing)	
Assembly metrics *		*Benchmark*
Consensus quality (QV)	56.9	*≥ 50*
*k*-mer completeness	100.0%	*≥ 95%*
BUSCO **	C:92.6%[S:86.9%,D:5.7%],F:1.8%,M:5.6%,n:2326	*C ≥ 95%*
Percentage of assembly mapped to chromosomes	98.06%	*≥ 95%*
Sex chromosomes	-	*localised homologous pairs*
Organelles	Mitochondrial genome: 333.39 kb Plastid genome: 163.28 kb	*complete single alleles*
Raw data accessions		
PacificBiosciences SEQUEL II	ERR6939259	
10X Genomics Illumina	ERR6688687, ERR6688689, ERR6688690, ERR6688688	
Hi-C Illumina	ERR6688691	
PolyA RNA-Seq Illumina	ERR9435018	
Genome assembly		
Assembly accession	GCA_934048045.1	
*Accession of alternate haplotype*	GCA_934047395.1	
Span (Mb)	351.6	
Number of contigs	142	
Contig N50 length (Mb)	16.6	
Number of scaffolds	86	
Scaffold N50 length (Mb)	33.8	
Longest scaffold (Mb)	42.79	

* Assembly metric benchmarks are adapted from column VGP-2020 of “Table 1: Proposed standards and metrics for defining genome assembly quality” from Rhie *et al*. (2021). ** BUSCO scores based on the eudicts_odb10 BUSCO set using v5.3.2. C = complete [S = single copy, D = duplicated], F = fragmented, M = missing, n = number of orthologues in comparison. A full set of BUSCO scores is available at https://blobtoolkit.genomehubs.org/view/CAKOHF01/dataset/CAKOHF01/busco.

**Table 2.** Chromosomal pseudomolecules in the genome assembly of *Polygonum aviculare*, dcPolAvic1.

INSDC accession	Chromosome	Length (Mb)	GC%
OW204023.1	1	42.79	39.0
OW204024.1	2	41.05	39.5
OW204025.1	3	36.28	39.5
OW204026.1	4	35.42	39.5
OW204027.1	5	33.83	39.0
OW204028.1	6	32.56	40.0
OW204029.1	7	31.96	39.0
OW204030.1	8	31.04	39.0
OW204031.1	9	31.0	39.5
OW204032.1	10	28.84	39.0
OW204033.1	MT	0.33	44.5
OW204034.1	Pltd	0.16	37.5

The estimated Quality Value (QV) of the final assembly is 56.9 with *k*-mer completeness of 100.0%, and the assembly has a BUSCO v5.3.2 completeness of 92.6% (single = 86.9%, duplicated = 5.7%), using the eudicots_odb10 reference set ( *n* = 2,326).

Metadata for specimens, barcode results, spectra estimates, sequencing runs, contaminants and pre-curation assembly statistics are given at https://links.tol.sanger.ac.uk/species/137693.

## Methods

### Sample acquisition, genome size estimation and nucleic acid extraction

A *Polygonum aviculare* (specimen ID KDTOL10108, ToLID dcPolAvic1) was picked by hand as a weed from a bed from Royal Botanic Gardens Kew (latitude 51.48, longitude –0.29) on 8 September 2020. The specimen was collected and identified by Maarten Christenhusz (Royal Botanic Gardens, Kew; collection no 9102) and then preserved by freezing at –80 °C. Herbarium voucher specimens collected from the same population as the sequenced specimen are lodged in the Herbarium of the Royal Botanic Gardens Kew (K) under accession number K001400691, and at the Royal Botanic Garden Edinburgh (E) ( https://data.rbge.org.uk/herb/E01152775).

The genome size was estimated by flow cytometry using the fluorochrome propidium iodide and following the ‘one-step’ method as outlined in Pellicer *et al*. (2021). For this species, the General Purpose Buffer (GPB) supplemented with 3% PVP and 0.08% (v/v) beta-mercaptoethanol was used for isolation of nuclei ( Loureiro *et al.*, 2007), and the internal calibration standard was *Petroselinum crispum* ‘Champion Moss Curled’ with an assumed 1C-value of 2,200 Mb ( Obermayer *et al.*, 2002).

Protocols developed by the Wellcome Sanger Institute (WSI) Tree of Life core laboratory have been deposited on protocols.io ( Denton *et al.*, 2023). The workflow for high molecular weight (HMW) DNA extraction at the WSI includes a sequence of core procedures: sample preparation; sample homogenisation, DNA extraction, fragmentation, and clean-up. In sample preparation, the dcPolAvic1 sample was weighed and dissected on dry ice ( Jay *et al.*, 2023). For sample homogenisation, leaf tissue was cryogenically disrupted using the Covaris cryoPREP ^®^ Automated Dry Pulverizer ( Narváez-Gómez *et al.*, 2023). HMW DNA was extracted using the Automated Plant MagAttract v2 protocol ( Todorovic *et al.*, 2023a). HMW DNA was sheared into an average fragment size of 12–20 kb in a Megaruptor 3 system with speed setting 30 ( Todorovic *et al.*, 2023b). Sheared DNA was purified by solid-phase reversible immobilisation ( Strickland *et al.*, 2023): in brief, the method employs a 1.8X ratio of AMPure PB beads to sample to eliminate shorter fragments and concentrate the DNA. The concentration of the sheared and purified DNA was assessed using a Nanodrop spectrophotometer and Qubit Fluorometer and Qubit dsDNA High Sensitivity Assay kit. Fragment size distribution was evaluated by running the sample on the FemtoPulse system.

RNA was extracted from leaf tissue of dcPolAvic1 in the Tree of Life Laboratory at the WSI using the RNA Extraction: Automated MagMax™ *mir*Vana protocol ( do Amaral *et al.*, 2023). The RNA concentration was assessed using a Nanodrop spectrophotometer and a Qubit Fluorometer using the Qubit RNA Broad-Range Assay kit. Analysis of the integrity of the RNA was done using the Agilent RNA 6000 Pico Kit and Eukaryotic Total RNA assay.

### Sequencing

Pacific Biosciences HiFi circular consensus and 10X Genomics read cloud DNA sequencing libraries were constructed according to the manufacturers’ instructions. Poly(A) RNA-Seq libraries were constructed using the NEB Ultra II RNA Library Prep kit. DNA and RNA sequencing was performed by the Scientific Operations core at the WSI on Pacific Biosciences SEQUEL II (HiFi), Illumina HiSeq 4000 (RNA-Seq) and Illumina NovaSeq 6000 (10X) instruments. Hi-C data were also generated from leaf tissue of dcPolAvic1 using the Arima2 kit and sequenced on the Illumina NovaSeq 6000 instrument.

### Genome assembly, curation and evaluation

Assembly was carried out with Hifiasm ( Cheng *et al.*, 2021) and haplotypic duplication was identified and removed with purge_dups ( Guan *et al.*, 2020). One round of polishing was performed by aligning 10X Genomics read data to the assembly with Long Ranger ALIGN, calling variants with FreeBayes ( Garrison & Marth, 2012). The assembly was then scaffolded with Hi-C data ( Rao *et al.*, 2014) using SALSA2 ( Ghurye *et al.*, 2019). The assembly was checked for contamination and corrected using the gEVAL system ( Chow *et al.*, 2016) as described previously ( Howe *et al.*, 2021). Manual curation was performed using gEVAL, HiGlass ( Kerpedjiev *et al.*, 2018) and Pretext ( Harry, 2022). The organelle genomes were assembled using MBG ( Rautiainen & Marschall, 2021).

A Hi-C map for the final assembly was produced using bwa-mem2 ( Vasimuddin *et al.*, 2019) in the Cooler file format ( Abdennur & Mirny, 2020). To assess the assembly metrics, the *k*-mer completeness and QV consensus quality values were calculated in Merqury ( Rhie *et al.*, 2020). This work was done using Nextflow ( Di Tommaso *et al.*, 2017) DSL2 pipelines “sanger-tol/readmapping” ( Surana *et al.*, 2023a) and “sanger-tol/genomenote” ( Surana *et al.*, 2023b). The genome was analysed within the BlobToolKit environment ( Challis *et al.*, 2020) and BUSCO scores ( Manni *et al.*, 2021; Simão *et al.*, 2015) were calculated.

Table 3 contains a list of relevant software tool versions and sources.

**Table 3.** Software tools: versions and sources.

Software tool	Version	Source
BlobToolKit	3.4.0	https://github.com/blobtoolkit/blobtoolkit
BUSCO	5.3.2	https://gitlab.com/ezlab/busco
FreeBayes	1.3.1-17-gaa2ace8	https://github.com/freebayes/freebayes
gEVAL	N/A	https://geval.org.uk/
Hifiasm	0.15.3-r339	https://github.com/chhylp123/hifiasm
HiGlass	1.11.6	https://github.com/higlass/higlass
Long Ranger ALIGN	2.2.2	https://support.10xgenomics.com/genome-exome/software/pipelines/latest/advanced/other-pipelines
MBG	-	https://github.com/maickrau/MBG
Merqury	MerquryFK	https://github.com/thegenemyers/MERQURY.FK
MitoHiFi	2	https://github.com/marcelauliano/MitoHiFi
PretextView	0.2	https://github.com/wtsi-hpag/PretextView
purge_dups	1.2.3	https://github.com/dfguan/purge_dups
SALSA	2.2	https://github.com/salsa-rs/salsa
sanger-tol/genomenote	v1.0	https://github.com/sanger-tol/genomenote
sanger-tol/readmapping	1.1.0	https://github.com/sanger-tol/readmapping/tree/1.1.0

### Wellcome Sanger Institute – Legal and Governance

The materials that have contributed to this genome note have been supplied by a Darwin Tree of Life Partner. The submission of materials by a Darwin Tree of Life Partner is subject to the **‘Darwin Tree of Life Project Sampling Code of Practice’**, which can be found in full on the Darwin Tree of Life website here. By agreeing with and signing up to the Sampling Code of Practice, the Darwin Tree of Life Partner agrees they will meet the legal and ethical requirements and standards set out within this document in respect of all samples acquired for, and supplied to, the Darwin Tree of Life Project. 

Further, the Wellcome Sanger Institute employs a process whereby due diligence is carried out proportionate to the nature of the materials themselves, and the circumstances under which they have been/are to be collected and provided for use. The purpose of this is to address and mitigate any potential legal and/or ethical implications of receipt and use of the materials as part of the research project, and to ensure that in doing so we align with best practice wherever possible. The overarching areas of consideration are:

•       Ethical review of provenance and sourcing of the material

•       Legality of collection, transfer and use (national and international)

Each transfer of samples is further undertaken according to a Research Collaboration Agreement or Material Transfer Agreement entered into by the Darwin Tree of Life Partner, Genome Research Limited (operating as the Wellcome Sanger Institute), and in some circumstances other Darwin Tree of Life collaborators.

## Data Availability

European Nucleotide Archive: *Polygonum aviculare*. Accession number PRJEB47313; https://identifiers.org/ena.embl/PRJEB47313 ( Wellcome Sanger Institute, 2022). The genome sequence is released openly for reuse. The *Polygonum aviculare* genome sequencing initiative is part of the Darwin Tree of Life (DToL) project. All raw sequence data and the assembly have been deposited in INSDC databases. The genome will be annotated using available RNA-Seq data and presented through the Ensembl pipeline at the European Bioinformatics Institute. Raw data and assembly accession identifiers are reported in Table 1.

## Author information

Members of the Royal Botanic Gardens Kew Genome Acquisition Lab are listed here: https://doi.org/10.5281/zenodo.4786680.

Members of the Royal Botanic Garden Edinburgh Genome Acquisition Lab are listed here: https://doi.org/10.5281/zenodo.4786682.

Members of the Plant Genome Sizing collective are listed here: https://doi.org/10.5281/zenodo.7994306.

Members of the Darwin Tree of Life Barcoding collective are listed here: https://doi.org/10.5281/zenodo.4893703.

Members of the Wellcome Sanger Institute Tree of Life Management, Samples and Laboratory team are listed here: https://doi.org/10.5281/zenodo.10066175.

Members of Wellcome Sanger Institute Scientific Operations: Sequencing Operations are listed here: https://doi.org/10.5281/zenodo.10043364.

Members of the Wellcome Sanger Institute Tree of Life Core Informatics team are listed here: https://doi.org/10.5281/zenodo.10066637.

Members of the Tree of Life Core Informatics collective are listed here: https://doi.org/10.5281/zenodo.5013541.

Members of the Darwin Tree of Life Consortium are listed here: https://doi.org/10.5281/zenodo.4783558.
