# Supplementary material for: The genome sequence of common knotgrass, Polygonum aviculare L. (Polygonaceae)
Source: Wellcome Open Res. 2024 Mar 1;9:112. [Version 1] doi: 10.12688/wellcomeopenres.21001.1 (PMC11294812; doi:10.12688/wellcomeopenres.21001.1)
Supplement: Peer review history

# Reviewer report: Reviewer response for version 1

**DOI:** 10.21956/wellcomeopenres.23236.r88619

**Published:** 2024-08-01

**Authors:** Lara Pereira

**Affiliations:** 1 Ecology and Evolutionary Biology, University of Sheffield, Sheffield, UK; 2 Plant Physiology, American Society of Plant Biologists, Rockville, USA

The article describes clearly and concisely the generation of a new high-quality assembly for *Polygonum aviculare*. The text is easy to follow, the tables and figures are clear and informative and all the relevant methods and datasets are described appropriately. 

 The genome assembly presented here is a valuable resource in the context of the Darwin Tree of Life Project, which aims to sequence all species in Great Britain. 

 I have no concerns about the quality of this report and the datasets published here, and I would therefore recommend this article for indexing.

Are sufficient details of methods and materials provided to allow replication by others?

Yes

Is the rationale for creating the dataset(s) clearly described?

Yes

Are the datasets clearly presented in a useable and accessible format?

Yes

Are the protocols appropriate and is the work technically sound?

Yes

Reviewer Expertise:

Plant genomics

I confirm that I have read this submission and believe that I have an appropriate level of expertise to confirm that it is of an acceptable scientific standard.

# Reviewer report: Reviewer response for version 1

**DOI:** 10.21956/wellcomeopenres.23236.r88617

**Published:** 2024-08-01

**Authors:** Manuel Spannagl

**Affiliations:** 1 PGSB - Plant Genome and Systems Biology, Helmholtz Zentrum München, Helmholtz Zentrum München-German Research Center for Environmental Health, Neuherberg, Germany

This paper describes the genome assembly of the herb common knotgrass. With an assembly size around 350Mb this represents a rather small plant genome. Sequencing and assembly follows the tree of life standards and is state-of-the-art.

 Noteworthy, analyses indicate that knotgrass has a hexaploid genome...the assembly presented here represents a single haplotype. If the knotgrass genome is indeed an allopolyploid it is interesting to see that the subgenomes seem to be similar enough to mainly collapse into a single haplotype...although contigs from a second haplotype are presented and available as well. It would be interesting to get an idea about the degree of similarity between the different (in theory three) haplotypes of knotgrass.

 The overall BUSCO score of around 93% complete is a little on the lower side...could this be attributed to the used BUSCO eudicots reference set and the place of Polygonum in the taxonomy?

Are sufficient details of methods and materials provided to allow replication by others?

Yes

Is the rationale for creating the dataset(s) clearly described?

Yes

Are the datasets clearly presented in a useable and accessible format?

Yes

Are the protocols appropriate and is the work technically sound?

Yes

Reviewer Expertise:

plant genomics, bioinformatics

I confirm that I have read this submission and believe that I have an appropriate level of expertise to confirm that it is of an acceptable scientific standard.
